# Supplementary figures and images for: Colibactin-induced damage in bacteria is cell contact independent
Source: mBio. 2024 Nov 22;16(1):e01875-24. doi: 10.1128/mbio.01875-24 (PMC11708049; doi:10.1128/mbio.01875-24)

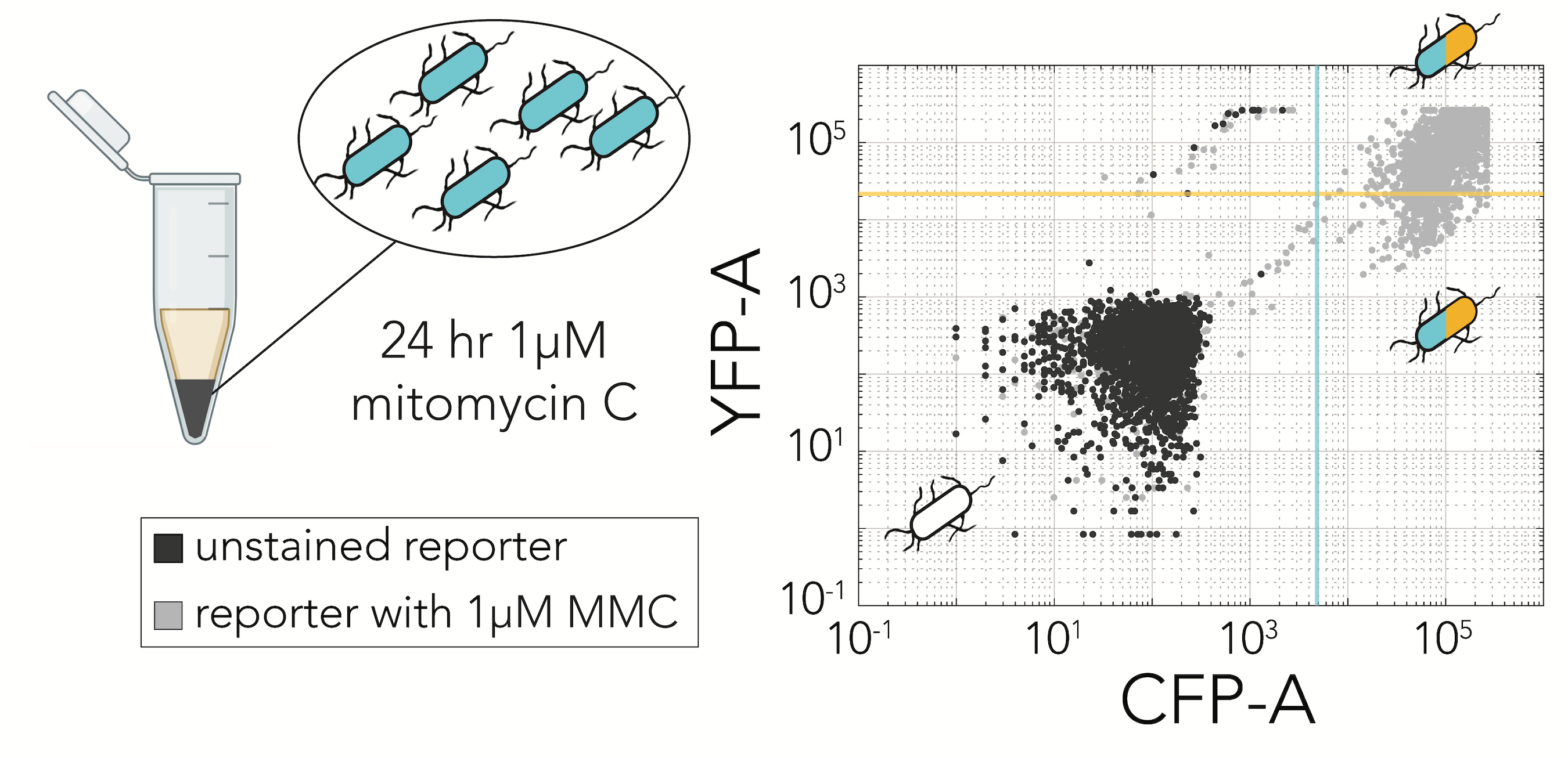

Supplement: Fig. S1 — Validation of the recA DNA damage reporter. [file mbio.01875-24-s0001.tif]

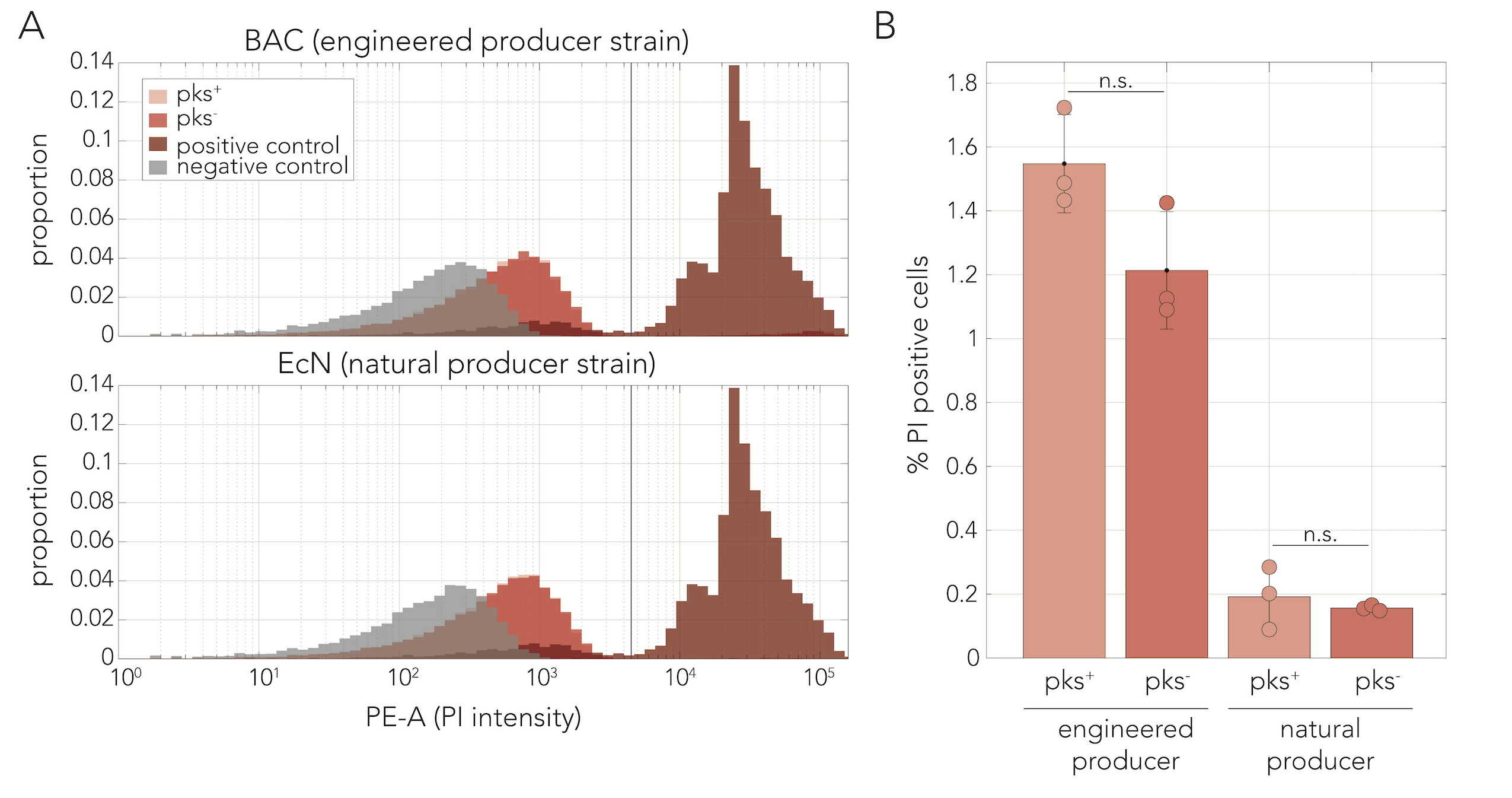

Supplement: Fig. S2 — Colibactin does not induce auto-lysis. [file mbio.01875-24-s0002.tif]

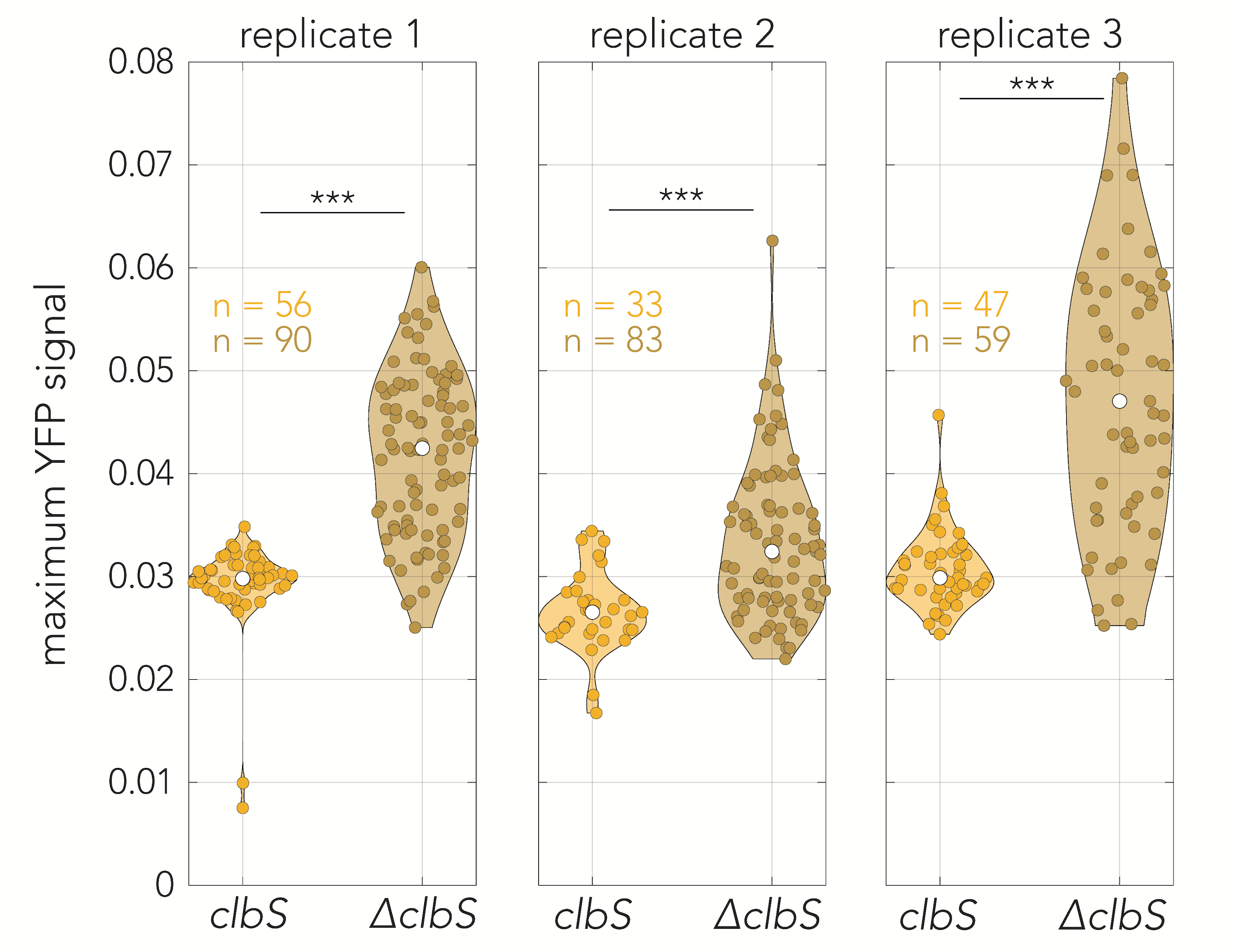

Supplement: Fig. S3 — ClbS protects cells from colibactin-induced DNA damage. [file mbio.01875-24-s0003.tif]

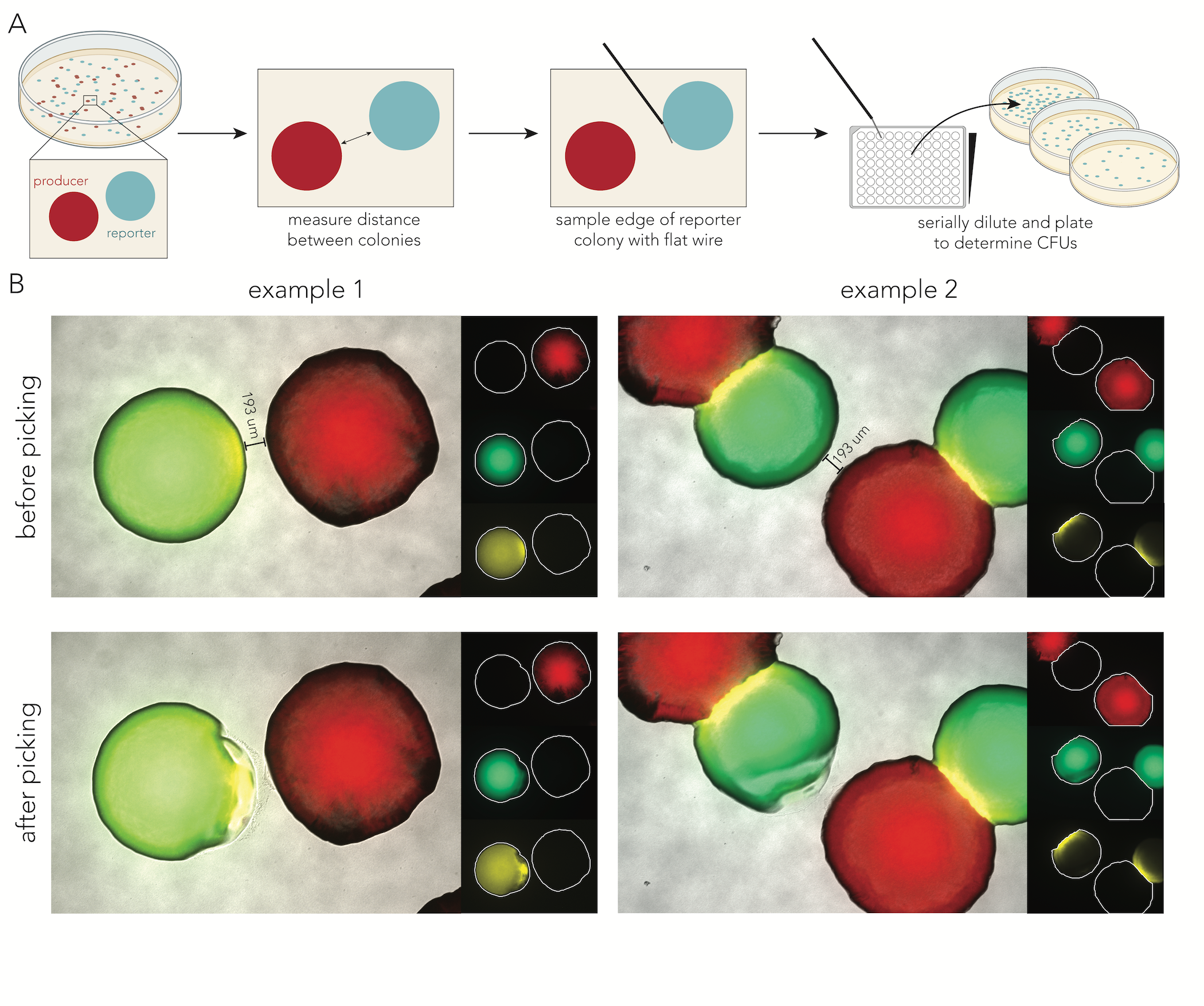

Supplement: Fig. S4 — Validation of strain purity in the edge of non-contacting colonies. [file mbio.01875-24-s0004.tif]

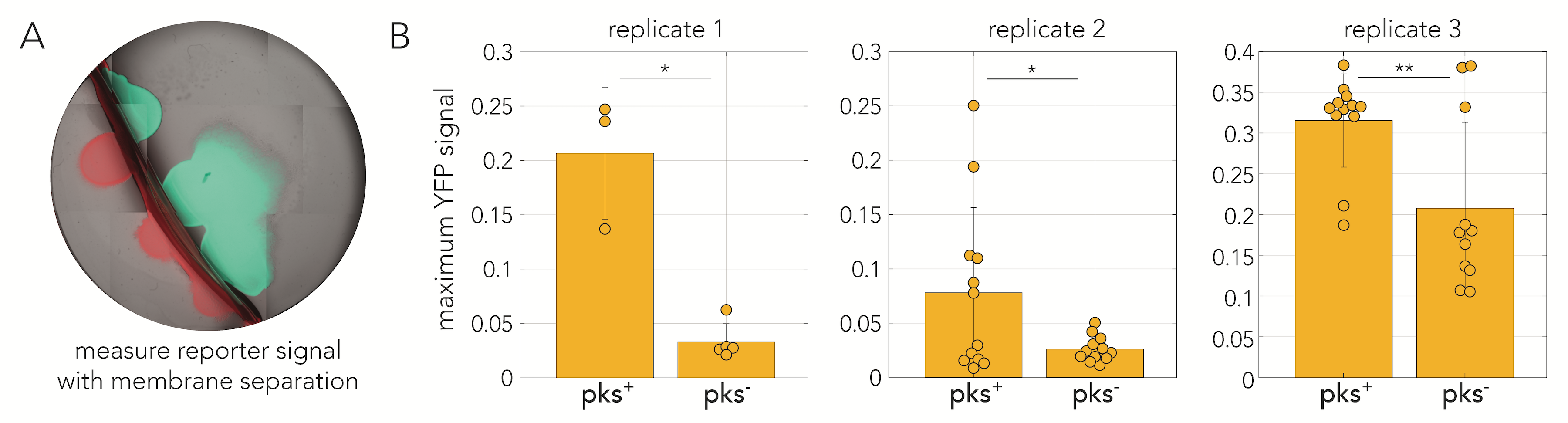

Supplement: Fig. S5 — Validation of contact independence with membrane separation. [file mbio.01875-24-s0005.tif]
